# From toilet to table: value-tailored messages influence emotional responses to wastewater products

**DOI:** 10.1186/s13068-021-01931-z

**Published:** 2021-03-30

**Authors:** Madeline Judge, Olivia de Hoog, Goda Perlaviciute, Nadja Contzen, Linda Steg

**Affiliations:** 1 Department of Psychology, Faculty of Behavioural and Social Sciences, University of Groningen, Grote Kruisstraat 2/I, 9712 TS Groningen, The Netherlands; 2 Department of Environmental Social Sciences, Eawag: Swiss Federal Institute of Aquatic Science and Technology, 8600 Duebendorf, Switzerland

**Keywords:** Wastewater products, Values, Emotions, Message framing, Acceptability, Intentions

## Abstract

**Background:**

Products made from recycled organic materials are an important part of a circular economy, but the question is whether they will be adopted by the public. Such products can elicit strong emotional responses and public resistance. As a case in point, we studied products made from sewage waste, such as recycled toilet paper, which can serve as material alternative to wood and plastic when making household items (e.g., tables). In an experimental study, we investigated the role of values in emotional responses to such wastewater products, and whether emotional responses were influenced by value-tailored messages. We expected that people would experience positive emotions towards products that supported their values, especially when the messages emphasised the benefits of these products for their values (e.g., when the products were presented as good for the environment). We presented participants with one of two messages describing wastewater products as having positive implications for either biospheric values (i.e. positive consequences for the environment) or hedonic values (i.e. positive consequences for personal enjoyment). We predicted that the relationship between values and positive emotions would be stronger when the messages emphasised the positive implications of wastewater products for one’s core values. Additionally, we predicted that emotions would be associated with acceptability and intentions to purchase the products.

**Results:**

The more strongly people endorsed biospheric values, the more positive emotions they reported towards wastewater products. As expected, this relationship was stronger when the environmental benefits of products were emphasised. Hedonic values were significantly but weakly associated with more negative and more positive emotions, and this did not depend on the message framing. However, we found that emphasising pleasurable benefits of wastewater products reduced positive emotions in people with weaker hedonic values. Positive and negative emotions were significantly associated with higher and lower acceptability of the products and intentions to purchase the products, respectively.

**Conclusions:**

Our findings have implications for the effective marketing of wastewater products. For people with strong biospheric values, emphasising the positive environmental consequences may promote wastewater products. Such biospheric messages do not seem to make the products less (or more) appealing for people with strong hedonic values, who do not generally have strong emotional responses to these products. We discuss the theoretical implications of our findings and avenues for future research.

**Supplementary Information:**

The online version contains supplementary material available at 10.1186/s13068-021-01931-z.

## Introduction

There is an urgent need for production and consumption systems worldwide to become more sustainable in order to combat climate change and environmental destruction [18]. An approach that is currently being widely promoted is to transition away from a linear economy (i.e. “take, make, use, dispose”) towards a more circular economy, which aims to reduce energy usage, reuse materials and minimise waste as much as possible [9]. As part of this transition, it is important to encourage consumer adoption of products made from recycled resources. However, recycled materials have the potential to elicit strong negative emotions in consumers. This is particularly the case for products made from sewage waste, such as recycled water (e.g., [10, 30, 39]) and materials made from recycled toilet paper (e.g., see the website, Cell-vation; https://www.cell-vation.com). Public resistance to wastewater products and other recycled products could impede a successful sustainable transition [18, 36]. It is therefore important to better understand the psychological bases of emotions elicited by these products, as well as the relationships between emotions and product acceptability and adoption.

Emotions play a key role in consumer satisfaction with products, as well as consumers’ willingness to purchase and use products (e.g., [27]), see also a special issue in the *Journal of the Association for Consumer Research* for a collection of recent research on this topic; [26]. Only recently has more attention been paid to the potential *negative* emotions elicited by environmentally friendly products, which can affect their uptake and use. For example, in one study, besides positive emotions, such as happiness and enthusiasm, plant-based plastic bottles elicited negative emotions, such as nervousness and worry, and both positive and negative emotions were related to purchase intentions [15]. Additionally, in another study, clothing made from recycled plastic elicited disgust, which was associated with lower purchase intentions [20]. Given that emotions can be related to consumers’ willingness to purchase and use products, it is important to understand what causes emotional responses to products. Thus far, this has received little attention in the research literature, so we aim to address this gap. We propose that emotional responses to products depend on the perceived implications of these products for people’s core *values* [24].

In the current research, we investigated how people who endorse different values to varying degrees respond emotionally to wastewater products. More specifically, we examined whether people who prioritised different values had different emotional responses to products made from recycled toilet paper, when different value-tailored messages were used to promote these products. We also investigated the relationships between emotions, acceptability of the products and intentions to purchase the products. In addition to providing theoretical insights into the role of values in emotional responses towards wastewater products, this research also offers practical insights into strategies for increasing positive emotions and reducing negative emotions, which can be related to their acceptability and adoption. That is, investigating which kinds of emotions are elicited by wastewater products, and the role of different values in these emotional responses, could be informative for considering how to effectively market these products (e.g., [17]; [19, 39]).

### Values and emotions towards sustainable innovations

Values can be defined as “concepts or beliefs that pertain to desirable end states or behaviours, transcend specific situations, guide selection or evaluation of behaviour and events, and are ordered by relative importance” ([32], p. 4). Values determine what people find important in life. Four types of values have been found to particularly influence attitudes and behaviours in the environmental domain, namely, altruistic (concern for other people), biospheric (concern for nature and the environment) egoistic (concern for money and resources) and hedonic (concern for personal pleasure) values [34]. For example, when evaluating energy production systems, individuals who strongly endorse biospheric values are more likely to attend to consequences for the environment, find them more important, and consider them more in decision-making [34, 35], whereas individuals who strongly endorse egoistic values are more likely to attend to personal consequences and find them more important [23].

It has been proposed that values can underlie emotional responses towards sustainable innovations [24]. Specifically, appraisal theory posits that people first evaluate a situation according to the characteristics that are goal relevant and goal (in)congruent (or alternatively, value relevant and value (in)congruent), which then, in turn, can elicit emotions [6]. Therefore, emotional responses to an innovation could depend on how people perceive the (in)congruence of the innovation’s specific characteristics with their core values [24]. For example, if one has strong biospheric values, then perceiving an innovation to have characteristics that support biospheric values (e.g., if the innovation produces less carbon emissions) could elicit positive emotions. In contrast, perceiving an innovation to have characteristics that conflict with biospheric values (e.g., if the innovation produces more waste) could elicit negative emotions.

Wastewater products are interesting for investigating the role of values in emotional responses to sustainable innovations. On the one hand, wastewater products may elicit positive emotions such as happiness and excitement because they benefit the environment. On the other hand, these products may also elicit negative emotions such as fear and disgust because they originate from sewage. As such, we suggest that wastewater products may have strong implications for two values in particular: biospheric values and hedonic values. Specifically, we expected that people with stronger biospheric values would experience more positive emotions and less negative emotions towards wastewater products, because these products are more environmentally friendly than conventional products. In contrast, we expected that people with stronger hedonic values would experience less positive emotions and more negative emotions towards wastewater products, because these products originate from sewage systems and may be seen as contaminated, non-pleasurable, and disgusting. These emotions are likely to be associated with preferences and choices. There is some initial evidence to support our reasoning. Specifically, research on behaviours that are environmentally friendly but potentially less pleasurable (e.g., taking shorter showers, eating less meat, owning fewer cars and not leaving electric devices on standby) has shown that endorsing biospheric values is positively related to engaging in such behaviours, whereas endorsing hedonic values is negatively related [34]. However, the role of emotions in these processes has not yet been explored.

### Tailoring messages to audiences

It may be possible to make product evaluations more positive by appealing to people’s values, via messages indicating that product has positive consequences for their values. Marketing messages are commonly used to advertise the benefits of products to consumers, in order to elicit more favourable responses and positive emotions. We argue, however, that the effectiveness of such messages is likely to depend on how these messages connect with the audience’s values. We propose that product communications (or message frames) that indicate positive implications for people’s core values may elicit more positive emotions towards the relevant product. Several studies have indeed found that value-tailored appeals aimed towards a specific audience tend to be more persuasive. For example, environmental campaigns (e.g., reducing plastic bottle use, or saving paper) are more convincing when the campaign messages are tailored to align with the audience’s biospheric or egoistic values [2], [37]. Most research thus far has not specifically examined the effects of *hedonic* message frames (i.e. a specific form of self-enhancement message). Thus, there is a need for more research on the role of emotions, as well as the impact of hedonic message frames. This might be particularly relevant for wastewater products, given that these products likely have negative implications for hedonic values, and could produce negative emotions.

Since wastewater products have implications for biospheric values, one obvious marketing strategy for wastewater products is to appeal to the audience’s biospheric values, by using biospheric message frames that highlight the environmental benefits of the products (e.g., that the products reduce waste). Hence, whereas people with strong biospheric values are likely to already experience positive emotions to wastewater products, this effect may be more pronounced when the positive impacts of these products are emphasised. Importantly, although stronger hedonic values could be associated with negative emotions towards wastewater products (given the implications of lower enjoyability), positive emotional responses could potentially be increased with hedonic message frames that highlight the personal pleasurable benefits of the product, such as its modern design. Thus, we tested the effects of framing wastewater products as having benefits for the environment (i.e. a biospheric message frame), or as having benefits for personal enjoyment (i.e. a hedonic message frame), for people who support biospheric and hedonic values to varying degrees.

### The current research

We first investigated whether emotional responses to wastewater products depend on people’s biospheric and hedonic values. We predicted that the stronger the audience’s biospheric values, the more positive (H1) and less negative (H2) emotions people would experience in response to wastewater products. Conversely, we predicted that the stronger the audience’s hedonic values, the less positive (H3) and more negative (H4) emotions people would experience in response to wastewater products. We then examined whether the effects change when the messages describe wastewater products as congruent with either biospheric or hedonic values. More specifically, we predicted that the relationship between biospheric values and positive emotions would be stronger for the biospheric message frame (e.g., emphasising these products reduce plastic waste and greenhouse gas emissions), relative to the hedonic message frame (e.g., emphasising that the products will enhance your interior and draw attention) (H5), and that the relationship between hedonic values and positive emotions will be stronger when the pleasurable benefits (versus environmental benefits) of the products were emphasised (H6). We did not make any directional hypotheses about the moderation effect for negative emotions. Lastly, we examined how emotions related to acceptability and intentions to purchase the products. We predicted that stronger positive (negative) emotions would be associated with higher (lower) acceptability of the products and intentions to purchase the products (H7). We tested these hypotheses in an online experimental study conducted in the Netherlands.

Thus far, most research on the acceptability of wastewater products has focused specifically on responses to *recycled water* (for a review, see [8]. In the current research, however, we focused on products made from wastewater *materials*. These are products made with cellulose fibres from toilet paper in wastewater, which during production can be mixed with other materials such as biodegradable bioplastic. These wastewater products can range from facade elements, asphalt, acetic acid, plant pots, telephone holders to table tops. The products are sustainable because they are made from recycled cellulose, saving natural resources and reducing emissions (e.g., see the website, Cell-vation; https://www.cell-vation.com). In addition, waste from these new products is also reduced, because the cellulose is biodegradable [1]. Hence, these products support the circular economy. The specific wastewater products studied in the current research were made from the recently developed material “Recell®”. To make Recell®, cellulose fibres from toilet paper are extracted from wastewater, and dried and sanitised so they are safe for use. The fibres are then turned into pallets or fluff, which is used to make various household products (e.g., a plant pot or a table top).

## Results

Means, standard deviations and bivariate correlations between all variables can be found in Additional file 1: Tables S1 and S2. We ran hierarchical linear regression analyses to test our hypotheses regarding the main effects of values on emotions, and the moderating role of message framing on the relationship between values and emotional responses to the products.^1^,^2^ For positive emotions (see Table 1), there were significant main effects of biospheric values, hedonic values and message framing for both products. As predicted, the more strongly that people endorsed biospheric values, the more positive emotions they reported in response to the products (supporting H1). The relationship was non-significant for negative emotions (see Table 2; not supporting H2). Conversely, the more strongly that people endorsed hedonic values, the more negative emotions towards the plant pot (partially supporting H4), *and* the more positive emotions towards both products (not supporting H3) they reported. In general, the biospheric frames elicited more positive emotions than the hedonic frames.

**Table 1:** Moderated regressions predicting positive emotions from values and framing

Predictors	Plant pot				Table top			
	*β*	*t*	*R*^*2*^	Δ *R*^*2*^	*β*	*t*	*R*^*2*^	Δ *R*^*2*^
Step 1			.14**	.14**			.12**	.12**
Biospheric values	.32**	5.94			.28**	5.25		
Hedonic values	.12*	2.24			.11*	2.08		
Framing	− .14**	− 2.73			− .14**	− 2.69		
Step 2			.17**	.03**			.15	.03**
Biospheric values	.31**	5.98			.28**	5.29		
Hedonic values	.13*	2.53			.13*	2.42		
Framing	− .14**	− 2.77			− .14**	− 2.73		
Biospheric values*Framing	− .14**	− 2.72			− .12*	− 2.30		
Hedonic values*Framing	.13*	2.54			.15**	2.74		

^*^p < .05, **p < .01. Values were mean centred prior to analyses. Framing: − 1 = biospheric, 1 = hedonic. For the plant pot, Step 2, *F change* (2,308) = 6.19, *p* = .002. For the table top, Step 2, *F change* (2,308) = 5.73, *p* = .004

**Fig. 1:**
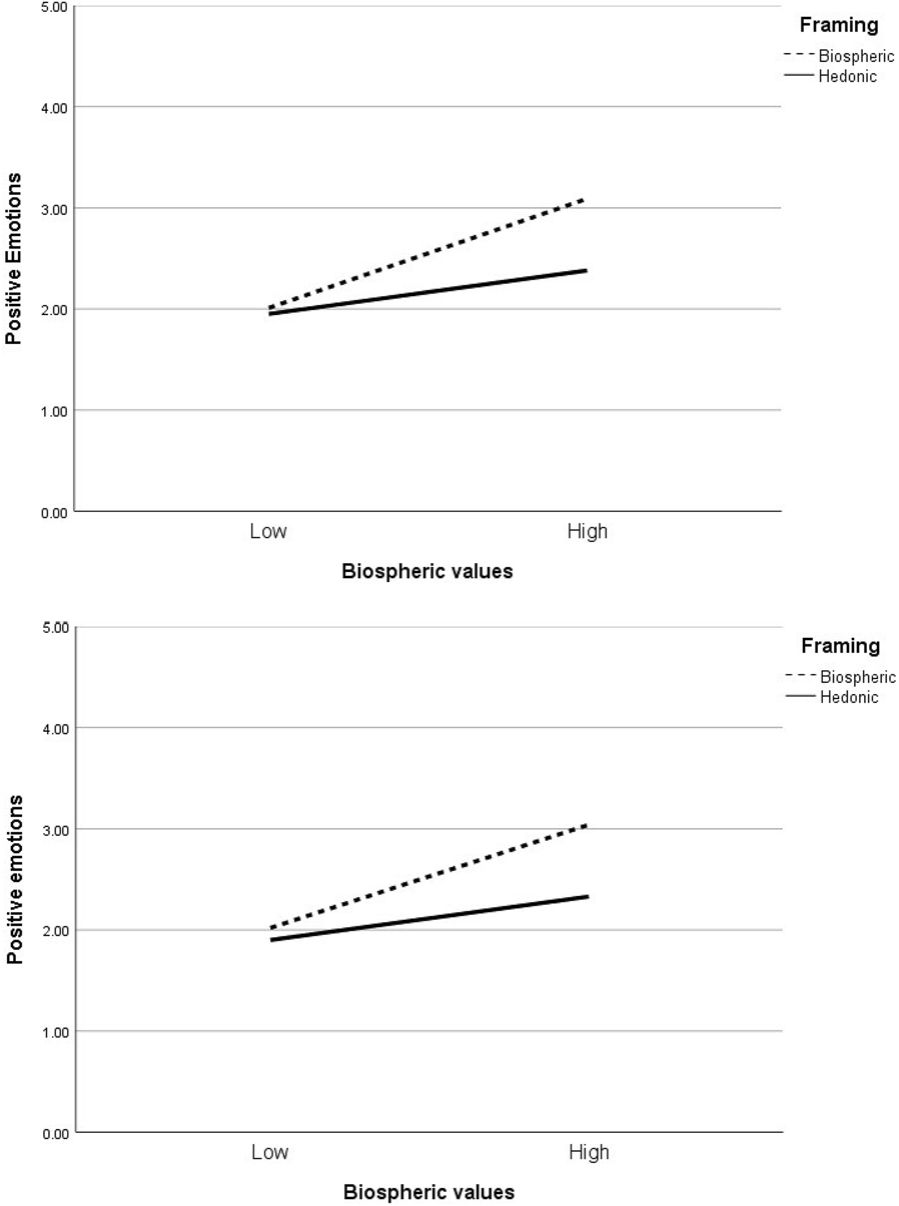
Interaction of biospheric values and framing on positive emotions. Top figure—plant pot; bottom figure—table top

As expected, these main effects were qualified by significant interactions between the values and the message framing for positive emotions. When the interaction terms were entered, there was a significant change in the explained variance, indicating a moderation effect. We conducted simple slopes analyses using PROCESS with 5,000 bootstrapped resamples [11], Model 1) to examine the interactive effects on positive emotions, run separately for biospheric values and hedonic values.^3^ For the plant pot, biospheric values were more strongly associated with positive emotions when the environmental benefits were emphasised, *b* = 0.39, *t*(314) = 6.27, *p* < 0.001, than when the pleasurable benefits were emphasised, *b* = 0.17, *t*(314) = 2.51, *p* = 0.01. Similarly, for the table top, the relationship between biospheric values and positive emotions was also stronger when the environmental benefits were emphasised, *b* = 0.37, *t*(314) = 5.43, *p* < 0.001, than when the pleasurable benefits were emphasised, *b* = 0.17, *t*(314) = 2.33, *p* = 0.02. These findings provide support for H5: the relationship between biospheric values and positive emotions appeared to be accentuated when the environmental rather than hedonic benefits of the products were emphasised (see Fig. 1).

**Fig. 2:**
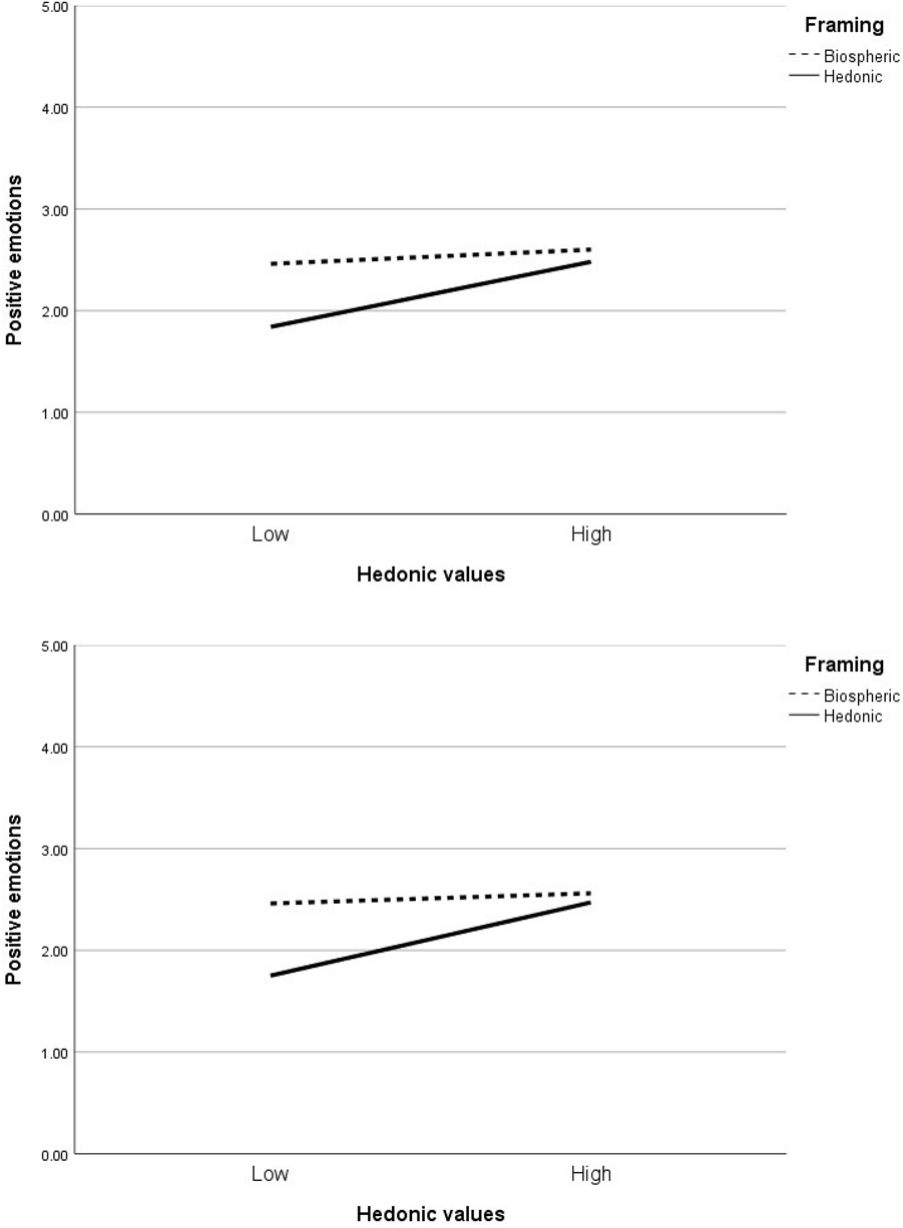
Interaction of hedonic values and framing on positive emotions. Top figure—plant pot; bottom figure—table top

Conversely, the effect of hedonic values on positive emotions was stronger when the pleasurable benefits were emphasised than when the environmental benefits were emphasised. Unexpectedly, however, the significant interaction between hedonic values and message frame seemed to be primarily driven by individuals with *weaker* hedonic values: individuals with relatively weak hedonic values tended to express less positive emotions when viewing the hedonic frames compared to the biospheric frames, whereas individuals with relatively strong hedonic values expressed a similar degree of positive emotions to both frames (see Fig. 2). More specifically, for the plant pot, weaker endorsement of hedonic values was associated with less positive emotions when the pleasurable benefits were emphasised, *b* = 0.25, *t*(314) = 3.31, *p* < 0.001, but not when the environmental benefits were emphasised, *b* = 0.06, *t*(314) = 0.93, *p* = 0.35. Similarly, for the table top, weaker endorsement of hedonic values was associated with less positive emotions when the pleasurable benefits were emphasised, *b* = 0.28, *t*(314) = 3.40, *p* < 0.001, but not when the environmental benefits were emphasised, *b* = 0.04, *t*(314) = 0.60, *p* = 0.55. Therefore, these findings do not seem to provide support for H6.

For negative emotions, there were no significant interactions between biospheric values and message framing, or between hedonic values and message framing, for either product (see Table 2).

**Table 2:** Moderated regressions predicting negative emotions from values and framing

Predictors	Plant pot				Table top			
	*β*	*t*	*R*^*2*^	Δ *R*^*2*^	*β*	*t*	*R*^*2*^	Δ *R*^*2*^
Step 1			.02^a^	.02^a^			.02	.02
Biospheric values	.01	.16			.07	1.21		
Hedonic values	.13*	2.33			.11	1.94		
Framing	.07	1.27			.05	.82		
Step 2			.03	.01			.03	.01
Biospheric values	.01	.23			.07	1.26		
Hedonic values	.14*	2.39			.11	1.96		
Framing	.07	1.26			.05	.81		
Biospheric values*Framing	.07	1.27			.07	1.24		
Hedonic values*Framing	.01	.22			− .004	− .07		

^*^p < .05, **p < .01. Values were mean centred prior to analyses. Framing: − 1 = biospheric, 1 = hedonic

^a^ *p* = .06. For the plant pot, Step 2, *F change* (2,308) = .87, *p* = .42. For the table top, Step 2, *F change* (2,308) = .77, *p* = .46

## Discussion

This research examined the role of values and value-tailored messages in emotional responses to wastewater products. Given the possible implications of wastewater products for both the environment and one’s personal comfort, we focused on the role of biospheric and hedonic values that reflect a concern with the environment and personal comfort, respectively. We predicted that individuals with stronger biospheric values would report more positive and less negative emotions in response to wastewater products, whereas individuals with stronger hedonic values would report less positive and more negative emotions. Additionally, we predicted that the relationships with positive emotions would depend on value-tailoring, that is, presenting the products as having positive implications for the relevant values. We hypothesised that value-tailored messages would increase positive emotions towards the products. We also examined whether emotions, in turn, were associated with acceptability of wastewater products and intentions to purchase wastewater products. Overall, the hypotheses were partially supported. We provide further explanation of the results separately for biospheric and hedonic values below.

### Biospheric values and emotions towards wastewater products

Biospheric values were associated with more positive emotions towards the products (i.e. supporting H1). Importantly, as expected, we found that this relationship was stronger when the product messages emphasised the positive environmental consequences of wastewater products (i.e. the positive implications for biospheric values; supporting H5). Thus, it appears that, in general, the stronger their biospheric values, the more positive emotions people have towards wastewater products, and this is even more likely when the benefits for the environment are emphasised in the product messages.

Interestingly, we did not find a significant relationship between biospheric values and less negative emotions towards wastewater products (not supporting H2). This could have been because our selected products did not elicit very strong negative emotions in the first place. Specifically, the low mean scores and low variance for the measures of negative emotions suggest that the specific products that we included may have not been ‘disgusting’ enough to generate sufficient variation in the negative emotion responses, or that the emotion terms selected to represent negative emotions were too strong for the products. For instance, 89% of participants chose “Not at all” when asked if they feel uncomfortable when imagining using the table top. Thus, the specific products chosen for the current research seem to have produced a floor effect for the measure of negative emotions. Previous research has found that people can be distressed about [39], and unwilling to consume [30] treated wastewater. However, the wastewater products included in the current research may not have been considered as disgusting as recycled water, because these products do not have to be ingested or come into contact with the body (see also, [13, 20]). Therefore, we would recommend that a wider range of products are included in future studies, particularly products that involve a high degree of physical contact or are used for oral consumption (e.g., drinking cups or straws).

### Hedonic values and emotions towards wastewater products

Hedonic values were weakly associated with negative emotions towards the plant pot, but not the table top (i.e. partially supporting H3). Hedonic values were also significantly associated with positive emotions (not supporting H4), especially when the product messages emphasised the pleasurable benefits of the products (i.e. the positive implications for hedonic values). Contrary to what we predicted in H6, however, this relationship seemed to be driven by *weaker* hedonic values. Specifically, weaker hedonic values were associated with *less* positive emotions when the pleasurable benefits were emphasised, relative to when the environmental benefits were emphasised, whereas stronger hedonic values were associated with similar levels of positive emotions in both message frames.

The relatively weak relationships between hedonic values and emotional responses towards wastewater products suggest that these kinds of products—or, at least, the products included in the current research—are probably not perceived as having major implications for hedonic values. This is unlikely to be due to different overall levels of endorsement of hedonic values in the sample, since both hedonic and biospheric values were endorsed by participants to a similar degree. The explanation that the current products did not have strong implications for hedonic values is also plausible because we found very low scores on negative emotions—which suggests that most people did not feel disgusted, anxious or uncomfortable in response to the products. Future research could include more disgusting products.

Another potential reason why hedonic values did not strongly predict emotions, is that the hedonic message frame may not have been relevant enough in this context. That is, the hedonic frames may have been generally less appealing than biospheric frames. For example, it could be the case that messages emphasising the environmentally friendliness of wastewater products are simply more convincing and intuitive (because this would be a primary reason to use such materials), whereas messages emphasising their pleasurable qualities may be seen as confusing, or even disingenuous (since these products might not be experienced as typically pleasurable in nature). Additionally, emphasising the origins of the materials could have had an unexpected effect of making the products seem more special and unique (given that products made from recycled toilet paper are relatively uncommon), whereas emphasising the pleasurable aspects of the products could have unexpectedly made the products seem more generic and conventional (i.e. just describing a product as ‘unique’ does not necessarily make it actually appear unique, because this is a very common marketing strategy). Alternatively, perhaps the hedonic frame may have immediately led people to consider the potential negative hedonic aspects of the products as well—although this explanation would still imply that stronger hedonic values would tend to have *less* positive emotions towards the products, which was not supported.

Unexpectedly, it appeared that individuals with weaker hedonic values, tended to express *less* positive emotions to the products when the hedonic frame was employed, relative to the biospheric frame, whereas individuals with stronger hedonic values did not differentiate strongly between the frames in their emotional responses. This suggests that when message frames are used that try to appeal to values that people find less important, these frames could potentially backfire. Future research should try to replicate and clarify the reasons for this finding. One possible explanation is that the hedonic message frame contained content less relevant to hedonic values (e.g., “drawing attention” could communicate a status motivation, which is more relevant for egoistic values). Additionally, while the biospheric frame primarily communicated implications for one main value, the hedonic message may have contained mixed content relevant to both hedonic *and* biospheric values. For example, even just stating that the products are made from biodegradable plastic and reused toilet paper could imply that they are good for the environment, in addition to the hedonic content implying that they are also good for the self, which may have reduced the clarity of the hedonic message. Some previous research has found that combined messages (e.g., incorporating both biospheric and egoistic frames; van den [37] are less effective than single-value appeals, showing that messages tailored towards a specific value can be more effective than messages that attempt to appeal to multiple values. Future research could also further investigate the effectiveness of combined product messages incorporating both hedonic and biospheric content.

In the current research, we did not see negative effects of the biospheric frame for individuals with strong hedonic values. This may indicate that people can believe that products can be *both* sustainable and pleasurable, and there is not necessarily a trade-off between hedonic values and pro-environmental behaviours (e.g., [38]. Yet, overall, the hedonic messages in the current research seemed to be less effective for eliciting positive responses to wastewater products than the biospheric messages.

The current findings make a unique contribution by being one of the first studies to investigate the role of hedonic values and hedonic messages in the context of consumer products that are likely to have negative implications for hedonic values, and elicit negative emotions. Most past research on value-congruent messaging and pro-environmental behaviours has not explicitly focused on hedonic values and messages, focusing instead on messages tailored towards either biospheric or egoistic values (e.g., [21],Van den [37], or not distinguishing hedonic messages from egoistic messages within a broader “self-enhancement” message frame (e.g., [4, 12]). For example, Herziger et al. [12] describe “wellbeing enhancement and stress reduction” as “selfish” interests related to egoistic values, although, arguably, these messages could also communicate self-care, which may be more relevant to hedonic values. Similarly, in the research by de Dominicis et al. [4], some of the content of the “self-enhancement” message frames appeared to be more hedonic in nature, such as people having fun at the beach. It can be quite difficult to disentangle egoistic and hedonic content when constructing messages that target solely one of these values. Indeed, even the hedonic frame in the current study could be viewed as containing egoistic elements (e.g., the reference to “drawing attention” could communicate a concern for one’s status). Thus, future research could pilot-test the specific value-tailored messages, to ensure that they are communicating the primarily targeted value. Hedonic values and hedonic message frames may have different effects on emotions towards sustainable products than egoistic values and egoistic message frames, for one because people may not perceive caring about the environmental benefits and the personal pleasure derived from such products as mutually exclusive, while they might do so for behaviours that are good for the environment and good for saving money. It appears that more research is needed to study the unique effects of hedonic values and hedonic message frames in the value-tailoring literature.

### Emotions, acceptability and intentions to purchase

We found consistent support for H7; positive emotions were associated with higher acceptability of wastewater products, and higher intentions to purchase wastewater products, whereas negative emotions were associated with lower acceptability of wastewater products, and lower intentions to purchase wastewater products. Emotions have been found to be highly intertwined with acceptability and are thus a relevant component in people’s reactions. In this paper, we have chosen to focus on investigating emotions, in particular, because these types of products could presumably cause strong emotions (e.g., disgust). H7 predicted that emotions would be associated with intentions to purchase; however, we did not hypothesise that emotions would act as a *mediator*, as this would suggest that emotions come first in a causal chain, before intentions. This causal chain may not necessarily be the case (e.g., [22, 31], and our design does not allow us to test causality or what comes first—emotions or intentions (rather, it is focused on testing the casual effects of value framing on emotions). Thus, we have focused on reporting the associations between emotions, acceptability and intentions. While we have run some exploratory moderated mediation analyses (see the Additional file 1), more experimental studies are needed to directly test the effects of emotions on intentions to purchase products.

The current findings are broadly consistent with previous research on the effects of message framing in responses to environmentally friendly products (e.g., [8, 15, 30]). Overall, our findings provide support that the implications of sustainable innovations for one’s strongly held values play a role in the elicitation of emotions and the acceptance of these innovations [24]. The findings are also consistent with previous research that has demonstrated that message framing can shape emotional reactions to wastewater products, like recycled water (e.g., [10]. We have extended this literature by demonstrating the key role of values in emotional responses to wastewater products.

### Limitations and future research

It is important to note that the current findings are based on a single study, and more research is needed to replicate and support these findings. A further limitation to this study is that we were not able to demonstrate whether the hedonic and biospheric message frames had beneficial effects when compared to a control condition (e.g., a condition containing very basic information about the product, with little value-related content). A control condition would allow for testing which of the message frames was primarily driving the effects on emotions (or if both messages were having an effect). We therefore recommend that future research includes a control condition. Another limitation is that we did not measure the *perceived* implications of the products for participant’s values. Thus, it cannot be concluded with certainty that people with stronger biospheric values had more positive emotions towards wastewater products *because* they perceived these products to have positive implications for their biospheric values (although the findings strongly suggest this). Future research could also include a measure of perceived value-congruence; that is, the extent to which participants think that a product has positive implications for their core values. Additionally, we were not able to conclude that the “hedonic” message frame was perceived as especially relevant to hedonic values. Future research could further test the effects of framing, particularly the hedonic framing; for example, by including clear value-relevant frames that have been pilot-tested. Alternative outcome measures also could be included in future research, such as relative willingness to pay (e.g., [29]), or behavioural measures; for example, by giving participants the opportunity to actually buy wastewater products. This would provide more evidence for the ecological validity of the findings and show that the manipulations are likely to have an impact on real-world behaviours.

Broader avenues for future research could include examining the role of other values, such as egoistic values or conservation values (i.e. valuing order and preserving the status quo; [33]). Previous research has found that purity and conservation values tend to be linked to trait disgust sensitivity (e.g., [14]; but see [5]), for a recent critique of this perspective). Therefore, it may be the case that ‘disgusting’ wastewater products evoke more negative emotions for people who hold strong conservation values than people who hold strong hedonic values.

Future research could also consider a more longitudinal perspective, rather than measuring reactions at a single time point. For example, disgust responses can change within individuals over time, a shift that can be intentional (e.g., via the motivated up-regulation or down-regulation of disgust to behave in ways that are more consistent with one’s values) or unintentional (e.g., from repeated exposure). For instance, there is recent evidence that people who work with meat (e.g., butchers, deli workers) exhibit adaptation of their disgust responses to meat over time [28]. In the current context, it is possible that individuals with stronger biospheric values may be motivated to down-regulate their disgust responses to wastewater products over time, because these products fit with their values. Also, arguably, the meaning of what is considered “disgusting” in society has changed a great deal over the course of history, and many culturally accepted innovations (e.g., fermented foods) could also be considered disgusting from a different cultural perspective [16]. Thus, disgust responses to circular economy products may be more amenable to change than is commonly assumed, and more research is needed in this area.

### Practical implications

The current findings provide useful insights for how to promote wide adoption of wastewater products. Although stronger biospheric values can lead to positive emotional reactions towards wastewater products, it may be important to clearly explicate the implications of the products for these values to consumers, in order to maximise the positive effect (consistent with the literature on value activation; e.g., [35]. Companies who are developing wastewater products may be reluctant to promote these products as environmentally friendly, since they may be concerned that this would increase the salience of the product’s origins and deter consumers from purchasing their products, particularly those with stronger hedonic values. The current findings suggest that this is not a major concern, participants holding strong hedonic values did not seem to have strong negative emotional responses to this type of wastewater product, even when the environmental benefits were emphasised. Thus, people do not seem to have large concerns about the implications of these products for their hedonic values. The findings also suggest that wastewater products are mostly appreciated for their environmentally friendly characteristics; in general, biospheric framing led to more positive emotions towards these products than hedonic framing, and there were more positive emotions elicited when the audience held strong biospheric values. Therefore, when audiences hold a variety of different values, it would likely be most beneficial to use biospheric message frames emphasising the positive benefits of the products for the environment. However, we should caution that we only included a small number of products and two types of values in the current research, and the hedonic frame did not produce the outcomes that we had expected. More research is needed to find out whether the findings would extend to other products, particularly products that require more physical contact, or how individuals respond based on their egoistic or altruistic values. Additionally, although the current research shows that biospheric frames can elicit positive emotions in people with strong biospheric values, it is less clear how we motivate people with low biospheric values to adopt wastewater products. This is another area for future research.

## Conclusions

To conclude, this research shows that biospheric values were consistently related to experiencing more positive emotions (but not less negative emotions) in response to wastewater products, whereas hedonic values did not tend to be (or were only weakly) related to positive and negative emotional responses. Stressing the positive environmental consequences could help promote wastewater products by increasing positive emotions, particularly for people with stronger biospheric values, and these messages seem not to make the products less appealing for people with stronger hedonic values. In contrast, stressing the pleasurable personal benefits of wastewater products does not seem to have much of a positive effect on emotions (and they could even backfire for those with weak hedonic values). Progressing this field of research has important practical implications for promoting the adoption of wastewater products and recycled products more generally, by demonstrating how effective messaging strategies can depend on the values and emotions of the specific target audience. If we can encourage more people to purchase such products, this could help promote the transition to a circular economy, which could ultimately contribute to saving natural resources and energy, and reducing greenhouse gas emissions and waste.

## Methods

### Participants

The study was run with a sample from the general Dutch public. It was estimated a priori that 263 participants were required to detect a small to medium effect size of 0.05 (similar to effect sizes reported for emotions in research with a similar methodology; [7], when using a power of 0.80, an alpha level of 0.05 and 5 predictors (biospheric values, hedonic values, framing, interaction biospheric values x framing and interaction hedonic values x framing). To allow for the removal of low-quality responses, we recruited 331 Dutch residents via thesistoolspro.com who received credit worth €1 to spend at a web store, Bol.com, upon completion of the questionnaire. We excluded 17 participants due to either failing an attention check (14 participants)^4^ or requesting to remove their data (three participants).^5^ The remaining sample comprised 314 participants (141 men, 173 women) who ranged in age from 16 to 88 (*M*_*age*_ = 53.80, *SD* = 15.36).^6^ Compared to the general Dutch population, fewer men and more women participated, and the sample was relatively more highly educated and had a higher income. Therefore, the sample was not completely representative of the Dutch population (see demographics in Additional file 1: Table S3).

### Research design

The research design was between-subjects with two conditions (a biospheric frame and a hedonic frame). Participants read short messages describing two products made with Recell®; a plant pot and a table top (all participants viewed both products). The first part of the text described how Recell® is made, accompanied by two pictures of the raw material (pallets and fluff) and a picture of each product (see full messages and pictures in Additional file 1). The second part of the text differed across the experimental conditions so that participants read one of the following messages (presented in English here, but in Dutch to participants).

*Biospheric frame* “Recell® and the products made from it are environmentally friendly. First, because less energy is needed for treating sewage water. Next, the plant pot is made from Recell® instead of oil-based plastic, which helps you to reduce plastic waste and greenhouse gas emissions. The table top is made from Recell® instead of wood, which helps saving valuable trees and reduces your carbon footprint.”

*Hedonic frame* The versatility of Recell® creates endless possibilities for creating modern interior objects. For the plant pot, Recell® is used to make granulate, which has a modern granite look that can fit nicely in the interior of your home. The refined table top made from Recell® will be a very unique piece of furniture in your house, completing your interior and drawing attention.”

### Procedure

The survey materials were prepared in English and then translated to Dutch (see messages and pictures in Additional file 1). The study was presented to participants as a study on the evaluation of different innovative products. We first measured participants’ values, and then participants were randomly assigned to either the biospheric framing condition or the hedonic framing condition, in which they read short messages describing two Recell® products emphasising either the environmentally friendly or pleasurable benefits of the products, respectively. After reading the product messages, participants reported their emotions towards the products, as well as their acceptability of, and intention to purchase the products.

#### Personal values

Personal values were measured with the shorter version of the Schwartz value scale (1992) developed by Steg et al. [34]. This scale consisted of 16 items measuring biospheric, hedonic, altruistic, and egoistic values. Participants rated the importance of each value as a guiding principle in their life, on a 9-point Likert scale from -1 (*opposed to my values*) to 7 (*of supreme importance*). The two subscales of interest were biospheric values (respecting the earth, unity with nature, protecting the environment, preventing pollution), *α* = 0.88 and hedonic values (pleasure, enjoying life, self-indulgent), *α* = 0.81.

#### Emotions

For each product (i.e. plant pot and table-top), participants indicated how they would feel if they used the product on a 6-point Likert scale from 0 (*not at all*) to 5 (*extremely strongly*). For positive emotions, we measured similar emotions as those in previous research on emotions towards drinking treated wastewater (e.g., [7],that is, happy, comfortable and excited, *α* = 0.86. We also measured three negative emotions, namely disgusted, anxious and uncomfortable,^7^*α* = 0.74.

#### Acceptability

Perceived acceptability of the products was measured with the question, “When thinking about the plant pot [table-top] just described, how would you evaluate this product on the following characteristics?”. Answers were given on four different 7-point bipolar scales (the numbers were not shown to participants), coded from − 3 (*very unacceptable*) to + 3 (*very acceptable*), − 3 (*very bad*) to + 3 (*very good*), − 3 (*very negative*) to + 3 (*very positive*) and − 3 (*very unnecessary*) to + 3 (*very necessary*), similar to the scales used by Perlaviciute et al. [25], *α* = 0.89.

#### Intention to purchase

Intention to purchase the products was measured with one item per product: “If you were to purchase a plant pot [table-top], how likely would you be to choose a plant pot [table-top] made from Recell® instead of a regular plant pot [table-top]?”. Answers were given on a 7-point scale from − 3 (*not at all likely*) to + 3 (*very likely*) separately for the plant pot and the table top (a single-item questionnaire is considered a valid measure of intention to purchase; [3]).

## Supplementary Information

**Additional file 1: Table S1.** Descriptive statistics for all variables indexed by framing condition. **Table S2.** Bivariate correlations between variables for the plant pot (above the diagonal) and the table top (below the diagonal). **Table S3.** Demographics in comparison to population percentages obtained by the central statistical office (CBS). **Figure S1.** Product description in biospheric framing condition. **Figure S2.** Product description in hedonic framing condition. **Figure S3.** Pictures of the plant pot, table top, pallets and fluff in both conditions.

## Data Availability

The datasets used and/or analysed during the current study are available from the corresponding author on reasonable request.

## Footnotes

1 We also ran the regressions separately for each individual interaction (e.g., where each regression model included just three predictors —one value type, the message framing and their interaction). However, the results did not change significantly, so we report the omnibus regressions here.

2 As noted by an anonymous reviewer, given that we have measured values, emotions, acceptability and intentions, it is technically possible to conduct moderated mediation analyses to examine whether values have indirect effects on acceptability/intentions via emotions, and whether these indirect effects are conditional on the message frame. However, these would just be exploratory analyses, since we did not experimentally manipulate emotions as the mediator. For interest, we conducted these exploratory analyses and reported them in the Additional file 1. In general, we found that values had significant indirect effects on acceptability/ intentions to purchase wastewater products via positive emotions, but these indirect effects were stronger (or only present) when the message frame matched the values.

3 An alternative way to break down the interactions is to compare the message frames at different levels of values. We have reported these alternative analyses in the Additional file 1.

4 The attention check was “If you read the questionnaire carefully, please click on the number six below” (participants had to select ‘6′).

5 The results did not change significantly when the participants who failed the attention check item were retained in the sample.

6 One respondent indicated an age of 270, which was considered as missing data.

7 The negative emotions items demonstrated high skewness (> -2) and kurtosis (> 3) scores (i.e., the vast majority of participants answered “Not at all” to these items), which means that the data violated some of the assumptions of the statistical tests, and so the findings for these variables should be interpreted with caution. The findings were similar when analysed with log-transformed variables.
