# Supplementary material for: From toilet to table: value-tailored messages influence emotional responses to wastewater products
Source: Biotechnol Biofuels. 2021 Mar 30;14:79. doi: 10.1186/s13068-021-01931-z (PMC8011086; doi:10.1186/s13068-021-01931-z)
Supplement: Supplementary file 1 — Additional file 1: Table S1. Descriptive statistics for all variables indexed by framing condition. Table S2. Bivariate correlations between variables for the plant pot (above the diagonal) and the table top (below the diagonal). Table S3. Demographics in comparison to population percentages obtained by the central statistical office (CBS). Figure S1. Product description in biospheric framing condition. Figure S2. Product description in hedonic framing condition. Figure S3. Pictures of the plant pot, table top, pallets and fluff in both conditions. [file 13068_2021_1931_MOESM1_ESM.pdf]

Additional File 1

Table S1.

Descriptive Statistics for All Variables Indexed by Framing Condition.

	Biospheric Frame (*n* = 161)		Hedonic Frame (*n* = 153)	
Variable	*M*	*SD*	*M*	*SD*
Biospheric values	4.80_a	1.41	4.93_a	1.38
Hedonic values	4.50_a	1.44	4.59_a	1.25
Positive emotions (plant pot)	2.53_a	1.23	2.23_b	1.16
Negative emotions (plant pot)	0.12_a	0.43	0.19_a	0.49
Acceptability (plant pot)	1.86_a	1.15	1.64_a	1.32
Intentions (plant pot)	1.06_a	1.63	0.56_b	1.59
Positive emotions (table top)	2.51_a	1.31	2.19_b	1.25
Negative emotions (table top)	0.14_a	0.44	0.20_a	0.59
Acceptability (table top)	1.73_a	1.29	1.56_a	1.30
Intentions (table top)	0.68_a	1.74	0.27_b	1.56

Note: Common subscripts denote no significant difference between the means

Table S2.

Bivariate Correlations between Variables for the Plant Pot (above the diagonal) and the Table Top (below the diagonal).

Variable	1	2	3	4	5	6
1. Biospheric values		.13*	.32**	0.03	.22**	.32**
2. Hedonic values	.13*		.15**	.14*	0.04	-0.04
3. Positive emotions	.29**	.14*		-0.08	.47**	.57**
4. Negative emotions	.09	.12*	-.07		-.34**	-.18**
5. Acceptability	.23**	.04	.52**	-.33**		.62**
6. Intentions	.27**	.02	.61**	-.21**	.61**	

Note. *$p < .05$, **$p < .01$.

Table S3

Sample Demographics in Comparison to Central Statistical Office (CBS) Percentages (n = 295)

Variable	Sample N	Sample percentage	CBS percentage
Gender			
Man	141	44.90%	49.60%
Women	173	55.10%	50.40%
Education			
Low Education	38	12.10%	31.10%
Intermediate Education	91	29.00%	37.80%
High Education	182	58.00%	29.60%
No Education/ prefer not to say/ different	3	1.00%	1.50%[a]
Yearly Net Household Income			
< 10.000	11	3.50%	4.90%
10.000 - 20.000	33	10.50%	25.90%
20.000 - 30.000	44	14.00%	32.40%
30.000 - 40.000	56	17.80%	21.20%
40.000 - 50.000	42	13.40%	8.90%
50.000 - 100.000	77	24.50%	6.00%
100.000 - 200.000	11	3.50%	0.60%
> 200.000	0	0.00%	0.20%
Prefer not to say	40	112.70%	-

Note. Education and income classification is based on the classification used by the CBS of the Netherlands. Gender is measured by the CBS in October 2018, education is the highest completed education of people 15 years and older measured by the CBS in May 2019 and yearly netto household income is measured by the CBS in December 2018.
[a]The CBS statistics refer here to the % of people indicating they do not know or the amount is unknown.

Alternative Breakdown of the Interaction Effects

An alternative way to break down the interaction effects is to compare the message frames at different levels of value endorsement. We have provided these alternative analyses below.

Biospheric values

For the plant pot, there were no differences between the message frames at low (−1 SD) levels of biospheric values, $b = -.02$, $t(314) = -.11$, $p = .91$. However, the biospheric frame elicited more positive emotions than the hedonic frame at high (+1 SD) levels of biospheric values, $b = -.65$, $t(314) = -3.66$, $p < .001$. Similarly, for the table top, there were no differences between the message frames at low (−1 SD) levels of biospheric values, $b = -.08$, $t(314) = -.40$, $p = .69$; however, the biospheric frame elicited more positive emotions than the hedonic frame at high (+1 SD) levels of biospheric values, $b = -.64$, $t(314) = -3.31$, $p < .001$.

Hedonic values

For the plant pot, the hedonic frame elicited significantly less positive emotions at low (−1 SD) levels of hedonic values, b = -.57, t(314) = -3.01, p < .001; however, there were no differences between the message frames at high (+1 SD) levels of hedonic values, b = -.05, t(314) = -.28, p = .78. Similarly, for the table top, the hedonic frame elicited significantly less positive emotions at low (−1 SD) levels of hedonic values, b = -.66, t(314) = -3.23, p < .001; however, there were no differences between the message frames at high (+1 SD) levels of hedonic values, b = -.02, t(314) = -.10, p = .92.

Additional Moderated Mediation Analyses

Using Model 7 of the PROCESS macro in SPSS with 5,000 bootstrapped resamples we conducted a series of moderated mediation analyses, with values as the predictor variable, emotions as the mediator, acceptability and intentions to purchase as the outcome variables, and message framing as the moderator. There were no significant indirect effects via negative emotions on acceptability or intentions to purchase (for either biospheric or hedonic values), so we focus on reporting the findings for positive emotions below.

Acceptability

For the plant-pot, there were significant conditional indirect effects of biospheric values on acceptability via positive emotions in both the biospheric frame, b = .16, 95%CI[.10, .23], and in the hedonic frame, b = .07, 95%CI[.01, .14]. The difference between these effects was significant, index of moderated mediation: -.10, 95%CI[-.18, -.03], indicating that the indirect effect of biospheric values on acceptability via positive emotions was stronger in the biospheric frame than in the hedonic frame. There was a significant conditional indirect effect of hedonic values on acceptability via positive emotions in the hedonic frame, b = .10, 95%CI[.04, .17], but not in the biospheric frame, b = .01, 95%CI[-.05, .06]. The difference between these effects was significant, index of moderated mediation: .09, 95%CI[.01, .18], indicating that the indirect effect of hedonic values on acceptability via positive emotions was significant in the hedonic frame, but not in the biospheric frame.

For the table top, there were significant conditional indirect effects of biospheric values on acceptability via positive emotions in both the biospheric frame, b = .16, 95%CI[.09, .24], and in the hedonic frame, b = .07, 95%CI[.004, .15]. However, the difference between these effects was non-significant; index of moderated mediation: -.09, 95%CI[-.19, .01], indicating that the indirect effect of biospheric values on acceptability via positive emotions did not depend on the message frame. There was a significant conditional indirect effect of hedonic values on

acceptability via positive emotions in the hedonic frame, *b* = .10, 95%CI[.03, .18], but not in the biospheric frame, *b* = .01, 95%CI[-.04, .06]. The difference between these effects was significant; index of moderated mediation: .09, 95%CI[.01, .19], indicating that the indirect effect of hedonic values on acceptability via positive emotions was significant in the hedonic frame, but not in the biospheric frame.

Intentions to purchase

For the plant-pot, there were significant conditional indirect effects of biospheric values on intentions to purchase via positive emotions in both the biospheric frame, *b* = .27, 95%CI[.16, .37], and in the hedonic frame, *b* = .11, 95%CI[.01, .23]. The difference between these effects was significant; index of moderated mediation: -.15, 95%CI[-.28, -.02] indicating that the indirect effect of biospheric values on intentions to purchase via positive emotions was stronger in the biospheric frame than in the hedonic frame. There was a significant conditional indirect effect of hedonic values on intentions to purchase via positive emotions in the hedonic frame, *b* = .16, 95%CI[.06, .27], but not in the biospheric frame, *b* = .01, 95%CI[-.07, .10]. The difference between these effects was significant; index of moderated mediation: .15, 95%CI[.02, .29] indicating that the indirect effect of hedonic values on intentions to purchase via positive emotions was significant in the hedonic frame, but not in the biospheric frame.

For the table top, there were significant conditional indirect effects of biospheric values on intentions to purchase via positive emotions in both the biospheric frame, *b* = .26, 95%CI[.15, .37], and in the hedonic frame, *b* = .12, 95%CI[.01, .24]. However, the difference between these effects was non-significant; index of moderated mediation: -.14, 95%CI[-.28, .01], indicating that the indirect effect of biospheric values on intentions to purchase via positive emotions did not depend on the message frame. There was a significant conditional indirect effect of hedonic values on intentions to purchase via positive emotions in the hedonic frame, *b* = .19, 95%CI[.08, .31], but not in the biospheric frame, *b* = .00, 95%CI[-.09, .10]. The difference between these effects was significant; index of moderated mediation: .19, 95%CI[.04, .34], indicating that the indirect effect of hedonic values on intentions to purchase via positive emotions was significant in the hedonic frame, but not in the biospheric frame.

Value-Tailored Framing Manipulation (in Dutch)

The product description in the biospheric framing condition is shown in Figure S1 and the product description in the hedonic framing condition is shown in Figure S2. The images are shown in Figure S3 (presented in both conditions)

Milieuvriendelijke producten van Recell®

De plantenpot en het tafelblad die u ziet zijn gemaakt van het materiaal Recell® en van een biologisch afbreekbaar bioplastic. Dit materiaal bestaat uit hergebruikte toiletpapier vezels. Om Recell® te maken worden toiletpapier vezels uit rioolwater gehaald (wat de waterkwaliteit verbetert) en vervolgens worden ze hygiënisch gemaakt, gedroogd en geschikt gemaakt voor gebruik. Van de vezels worden vervolgens korrels of pluizen gemaakt (zie beelden). Deze korrels en pluizen zijn geschikt om diverse producten van te maken.

Recell® en de producten die ervan gemaakt worden, zijn milieuvriendelijk. Allereerst, omdat er minder energie nodig is voor het behandelingsproces dan normaal gebruikt wordt om rioolwater te behandelen. Daarnaast is de plantenpot gemaakt van Recell® in plaats van plastic op oliebasis, wat bijdraagt aan de reductie van plastic afval en de uitstoot van broeikasgassen. Het tafelblad is gemaakt van Recell® in plaats van hout, wat helpt waardevolle bomen te besparen en wat uiteindelijk uw negatieve impact op het milieu kan verkleinen.

Figure S1. Product description in biospheric framing condition.

Moderne interieur producten van Recell®

De plantenpot en het tafelblad die u ziet zijn gemaakt van het materiaal Recell® en van een biologisch afbreekbaar bioplastic. Dit materiaal bestaat uit hergebruikte toiletpapier vezels. Om Recell® te maken worden toiletpapier vezels uit rioolwater gehaald (wat de waterkwaliteit verbetert) en vervolgens worden ze hygiënisch gemaakt, gedroogd en geschikt gemaakt voor gebruik. Van de vezels worden vervolgens korrels of pluizen gemaakt (zie beelden). Deze korrels en pluizen zijn geschikt om diverse producten van te maken.

Recell® is zeer veelzijdig en biedt eindeloze mogelijkheden voor het creëren van moderne interieur objecten. Voor deze plantenpot wordt Recell® gebruikt om granulaat van te maken, wat een moderne granietachtige uitstraling heeft en mooi in het interieur van uw huis kan passen. Dit verfijnde tafelblad gemaakt van Recell® zal een heel uniek meubelstuk in uw huis zijn wat uw interieur compleet kan maken en de aandacht trekt.

Figure S2. Product description in hedonic framing condition.

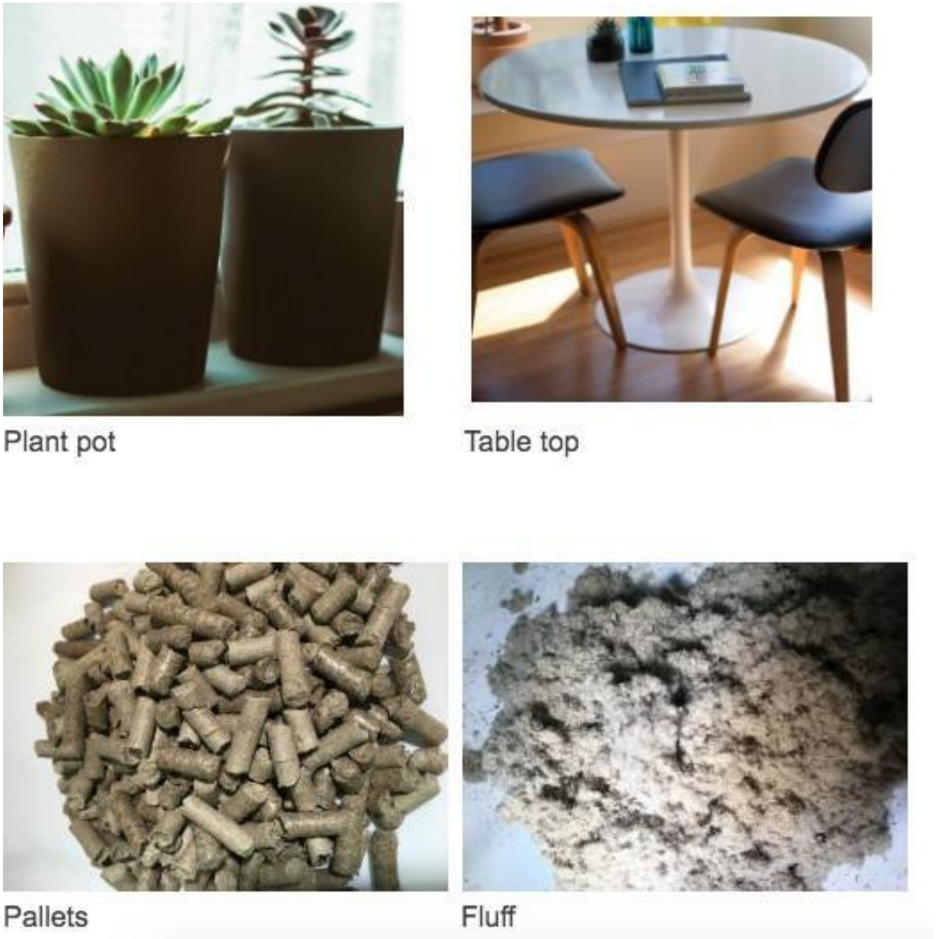

Figure S3. Pictures of the plant pot, table top, pallets and fluff in both conditions.
